# Chlorophyll *a* fluorescence lifetime reveals reversible UV-induced photosynthetic activity in the green algae *Tetraselmis*

**DOI:** 10.1007/s00249-015-1092-z

**Published:** 2015-11-04

**Authors:** Arne S. Kristoffersen, Børge Hamre, Øyvind Frette, Svein R. Erga

**Affiliations:** Department of Physics and Technology, University of Bergen, P.O. Box 7803, 5020 Bergen, Norway; Department of Biology, University of Bergen, P.O. Box 7803, 5020 Bergen, Norway

**Keywords:** Fluorescence lifetime, UV stress, Photosynthesis, Chlorophyll

## Abstract

The fluorescence lifetime is a very useful parameter for investigating biological materials on the molecular level as it is mostly independent of the fluorophore concentration. The green alga *Tetraselmis* blooms in summer, and therefore its response to UV irradiation is of particular interest. In vivo fluorescence lifetimes of chlorophyll *a* were measured under both normal and UV-stressed conditions of *Tetraselmis*. Fluorescence was induced by two-photon excitation using a femtosecond laser and laser scanning microscope. The lifetimes were measured in the time domain by time-correlated single-photon counting. Under normal conditions, the fluorescence lifetime was 262 ps, while after 2 h of exposure to UV radiation the lifetime increased to 389 ps, indicating decreased photochemical quenching, likely caused by a damaged and down-regulated photosynthetic apparatus. This was supported by a similar increase in the lifetime to 425 ps when inhibiting photosynthesis chemically using DCMU. Furthermore, the UV-stressed sample was dark-adapted overnight, resulting in a return of the lifetime to 280 ps, revealing that the damage caused by UV radiation is repairable on a relatively short time scale. This reversal of photosynthetic activity was also confirmed by ${F}_\mathrm{V}/{F}_\mathrm{M}$ measurements.

## Introduction

Both UV-A (315–400 nm) and particularly UV-B (280–315 nm) radiation reduce the growth rate and photosynthetic activity of phytoplankton and algae (Hessen et al. 1997; Holzinger et al. 2006; Holzinger and Lütz 2006). Some green algae exhibit a higher tolerance for UV radiation compared to other plants, with one study showing that for the unicellular freshwater green alga *Micrasterias denticulata*, only wavelengths lower than 284 nm were harmful to cells at the ultrastructural level (Meindl and Lütz 1996). Induction of increased secondary metabolites as a response to enhanced UV-B radiation is often observed in plants, where aromatic amino acids and phenolic compounds (flavonoids) are most common in aquatic algae and terrestrial vascular plants, respectively (Rozema et al. 1997, 2002). The photochemical efficiency of photosystem (PS) II in the red macroalgae *Palmaria palmata* and *Odonthalia dentata* has been shown to decrease to about one-third of the initial value under UV stress as well as experiencing damage to the fine structure of the photosynthetic apparatus, such as lumen dilatations and alterations of the outer membranes (Holzinger et al. 2006).

**Fig. 1:**
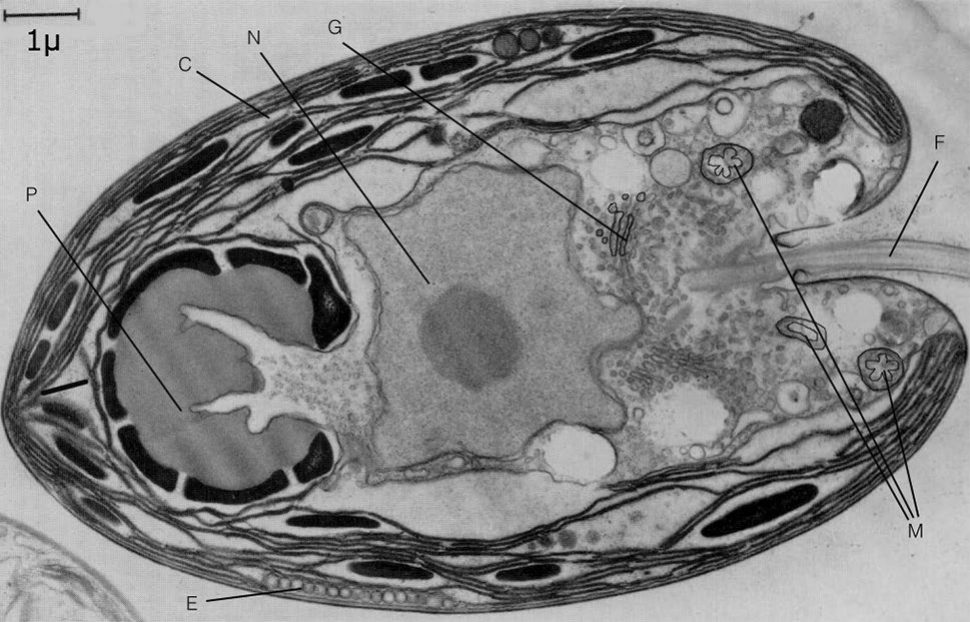
A section of a *Tetraselmis* cell showing the disposition of the nucleus (*N*), Golgi bodies (*G*), mitochondria (*M*), cup-shaped chloroplast (*C*), pyrenoid (*P*), eyespot (*E*) and flagella (*F*). Redrawn from (Manton and Parke 1965)

The genus *Tetraselmis* is in the class Prasinophyceae, which are free-living, flagellated green microalgae. *Tetraselmis* has adapted to a great variety of growth conditions, occurs in marine, brackish and freshwater habitats, and blooms in summer when UV irradiance is highest. The cells have a diameter of 8–10 ${\upmu} {\rm m}$ and are characterised by four equal flagella and a four-lobed chloroplast, which contains a stigma at its posterior end, and the exterior is covered by small organic scales (Fig. 1).

With respect to the functioning of the photosynthetic apparatus, the structural organisation of the thylakoids within the chloroplast is of great importance: it has been shown that the thylakoids in *T. subcordiformis* are organised into extended bands of usually 2–7 (sometimes more) appressed thylakoids, with an even distribution of PSI and PSII on all thylakoid membranes (Song and Gibbs 1995). This is different from higher plants, where the chloroplast thylakoids are usually organised into distinct grana and stroma lamella, and PSI and PSII are spatially separated in the thylakoid membrane (Taiz and Zeiger 2006). Another characteristic property of *Tetraselmis* species is their high chlorophyll (Chl) *b* content within the PSI, giving rise to Chl *a:b* ratios $\le$1.2 and a total of 800–2500 Chl *a* + *b* molecules per PSI, which is high compared to the 300–400 for higher plants (Smith and Alberts 1991).

The light harvesting complex (LHC) responsible for collecting light energy for use in photosynthesis is highly dynamic and able to alter its function in response to changes in the environment (Ruban et al. 2012). Light that is absorbed when the capacity for photosynthesis is saturated is a significant hazard to PSII since it can lead to the formation of highly reactive intermediates and by-products that can cause photo-oxidative damage and as a consequence inhibit photosynthesis (Niyogi 1999). More specifically, excess light causes a decrease in the thylakoid lumen pH and an increase in the reduction state of the plastoquinone pool and thiols in the chloroplast as well as the production of various reactive oxygen species (ROS)—mainly hydrogen peroxide ($\mathrm{H_{2}O_{2}}$) and singlet oxygen (Li et al. 2009). The generation of ROS is especially problematic as the production of one ROS can lead to the production of another. Excess light conditions promote the formation of the triplet excited state Chl and thus lead to the generation of a singlet oxygen, typically positioned at the reaction centre of PSII (Krieger-Liszkay 2005). Excess light also alters the synthesis rate of chloroplast-encoded proteins, such as the D1 protein of PSII (Shapira et al. 1997). A current model for higher plants is that when the thylakoid lumen pH decreases, a synthesis of zeaxanthin via a xanthophyll cycle and protonation of a PSII protein, PsbS, is activated, which transduces a conformational change to specific Chl- and carotenoid-binding light-harvesting complex proteins. For green algae, recent work has shown that the transcript levels of both genes encoding PsbS were strongly increased when the cells were deprived of nitrogen (Miller et al. 2010), suggesting that PsbS in green algae has either a role unrelated to photoprotection or is required for more efficient photoprotection under nitrogen deprivation. Dissipation of excess excitation energy of singlet state Chl was previously believed to occur by a charge-transfer mechanism involving a carotenoid radical cation (Ahn et al. 2008) and/or by Chl-to-carotenoid energy transfer (Ruban et al. 2007); however later experiments indicated that this is rather due to Chl–Chl interactions (Müller et al. 2010). Besides this, cyclic electron flow has been found to be important for the protection of PSI against high light stress (Roach and Krieger-Liszkay 2012). It has also been suggested that the Mehler reaction plays a role in the control of the activity of PSII, because thylakoid acidification is necessary in the formation of energy-dissipating traps in the antenna of PSII, which decrease the exciton pressure of the reaction centre (Heber 2002).

Chl fluorescence has been established as a sensitive indicator of the photosynthetic capacity in vivo (Kolber and Falkowski 1993; Falkowski and Kolber 1995; Schreiber et al. 1995), and it has been shown that exposure to UV radiation in the laboratory results in a sharp reduction of dark-adapted variable fluorescence followed by a gradual recovery after the samples were returned to the low irradiance of white light (Dring et al. 1996a, b). For the red macroalgal genus *Gelidium* such a pattern was confirmed for the sun-adapted species *G. latifolium*, while the shade-adapted species *G. sesquipedale* did not recover after exposure to UV radiation (Gómez and Figueroa 1998).

The fluorescence lifetime of Chl is an even more sensitive parameter compared to fluorescence intensity as it is mostly independent of the pigment concentration (Lakowicz 1999) and is directly affected by the molecular environment. The fluorescence lifetime is the average time a molecule spends in the excited state before returning to a lower state by de-excitation. There are many processes that serve as ways for the Chl molecules to return to a lower energy state. Most prominent are fluorescence, Förster resonance energy transfer and photochemistry, although binding interactions, heat dissipation via internal conversion, intersystem crossing from the Chl singlet state to the Chl triplet state, and transfer of energy from the excited triplet state of Chl to the ground state of oxygen are possible de-excitation processes (Lakowicz 1999; Papageorgiou and Govindjee 2004). All of these are in direct kinetic competition, which means that the Chl fluorescence lifetimes can be used to monitor many non-fluorescent reactions of photosynthesis. De-excitation by photochemistry is called qP, while all the other pathways are collectively referred to as non-photochemical quenching (NPQ). If the intrinsic rate of fluorescence is constant, changes in the fluorescence emission caused by NPQ directly reveal the quantitative properties of photosynthesis (Papageorgiou and Govindjee 2004). Furthermore, NPQ is classified into at least three types: (1) the pH-regulated energy-dependent quenching (qE), which occurs through charge separation at the reaction centre and is regulated by the breakdown of pH gradients (ΔpH). This takes place on the time scale of seconds to minutes, requiring the build-up of a trans-thylakoid proton gradient, and involves the xanthophyll cycle (Demmig-Adams and Adams 1996; Yamamoto 2006); (2) state transition type quenching (qT), which involves movement of light harvesting complexes (LHCs). This typically decreases the PSII antenna size and increases the PSI antenna size. This is the most important NPQ component in green algae, but does not contribute significantly under light saturated conditions. This type of quenching occurs on the time scale of 5–10 min; (3) photoinhibitory quenching (qI) is involved in long-term down-regulation of PSII. Under prolonged and severe stress, qE is replaced by a slow quenching, which is much less characterised compared to qE and qT possibly because of a mix of photoprotection and photodamage (Müller et al. 2001). The qI has been found to take place on a time scale of 30–60 min (Nilkens et al. 2010). A fourth, zeaxanthin (Zx)-depending NPQ process, termed qZ, has also been recently described. The formation (rise time 10–15 min) and relaxation (relaxation time 10–15 min) of qZ are correlated with both the synthesis and epoxidation of Zx and not related to qE, qT or qI (Nilkens et al. 2010). The relaxation of NPQ in green algae is strictly dependent on the removal of $\Delta$pH, and the maximal NPQ has been found to be much less dependent on Zx in green algae compared to higher plants (Jahns and Holzwarth 2012). All transitions have been found to be reversible; however, in the case for the slow component qI, data are not consequently supportive, as it was recently found to be almost irreversible (Nilkens et al. 2010), and previously it was reported to be slowly reversible (Müller et al. 2001). Traditionally, qI has been defined as reflecting all processes involved in a slow down-regulation, inhibition or damaging of PSII; however, it is likely that several different processes contribute to this traditional qI term, such as PSII inactivation due to the inhibition of either an acceptor or donor, or UV-B-related inactivation of PSII (Jahns and Holzwarth 2012).

Recent studies have shown from in vivo measurements that there are (at least) two lifetime components of Chl *a*. Slightly different lifetimes have been measured; however it is generally agreed that the main component (with the highest relative amplitude) is between 170 and 305 ps (Broess et al. 2009; Iwai et al. 2010; Amarnath et al. 2012; Kristoffersen et al. 2012). An ultra-short component at about 65 ps is attributed to PSI reaction centres. Closing the reaction centres by infiltration of DCMU (and thus shutting down photochemistry) has been shown to increase the fluorescence lifetime of the green algae *H. pluvialis* to around 420 ps (Kristoffersen et al. 2012), which is in accordance with the before-mentioned consequence: removing a de-excitation pathway results in an increased fluorescence lifetime. A second (slower) lifetime component often varies between 0.7 and 2 ns and is usually attributed to charge stabilisation and recombination in PSII reaction centres as well as closed or damaged reaction centres (Gilmore et al. 1995; Miloslavina et al. 2009; Holzwarth et al. 2009; Johnson and Ruban 2009).

Another non-invasive technique to study oxygenic photosynthetic organisms is the analysis of the kinetics of Chl *a*. When a dark-adapted photosynthetic sample is illuminated, the Chl *a* fluorescence intensity changes. This process has been termed the fluorescence transient, fluorescence induction or Kautsky effect (Kautsky and Hirsch 1931). Higher plants and algae under continuous light have a quickly increasing phase (less than a second) and a slowly decreasing phase (several minutes). The fast phase has been labeled the OJIP transient, with O (for origin) represents the initial minimum intensity level, J and I are intermediate intensities, and P is the intensity peak. Changes in the fluorescence intensity during the OJIP phase can be related to the reduction of the electron acceptors of the entire photosynthetic electron transport chain (Papageorgiou and Govindjee 2004). From the OJIP measurements it is most common to use the minimum fluorescence $F_\mathrm{o}$ and maximum fluorescence $F_\mathrm{M}$ to calculate the ratio $\dfrac{F_\mathrm{M}}{F_\mathrm{o}}$, which is usually around 5–6 (for plant leaves) (Björkman and Demmig 1987), and the ratio $\dfrac{F_\mathrm{V}}{F_\mathrm{M}}=\dfrac{F_\mathrm{M}-F_\mathrm{o}}{F_\mathrm{M}}$, which is used as a stress indicator and has been related to the maximum quantum yield of primary PSII photochemistry (Papageorgiou and Govindjee 2004; Stirbet and Govindjee 2011). For higher plants the latter ratio usually has a value in the range of 0.78–0.84 (Björkman and Demmig 1987), while it is slightly lower for microalgae, usually ranging from 0.6 to 0.7 (Parkhill et al. 2001; Guihéneuf et al. 2010; White et al. 2010).

## Materials and methods

### Instrumentation

A Ti:sapphire (Coherent Chameleon Ultra) laser generating femtosecond pulses (pulse width 140 fs) operating at an 80-MHz repetition rate (12.5 ns between pulses) was used to achieve two-photon excitation. The laser is tuneable from 690 to 1040 nm, with an average output power of about 3 W at the 860-nm wavelength used in the present study. An electro-optical modulator controlled the intensity of the laser beam, which was guided by mirrors into a laser scanning microscope (Leica TCS SP5) and focussed by a water immersion 63$\times$ objective with a numerical aperture of 1.2 and a working distance of 0.22 mm. All samples were scanned at a line frequency of 400 Hz and fluorescence was detected by a built-in photomultiplier tube (PMT) detector with a detection range from 400 to 800 nm. Line, frame and pixel clock signals were generated and synchronised by another PMT detector (Hamamatsu R3310-02) and linked via a time-correlated single-photon counting (TCSPC) imaging module (SPC-830, Becker-Hickl, Berlin, Germany) to generate fluorescence lifetime raw data. The UV spectrum from the irradiating lamps were measured using a Ramses radiometer (Trios, Oldenburg, Germany).

### Samples and preparation

**Fig. 2:**
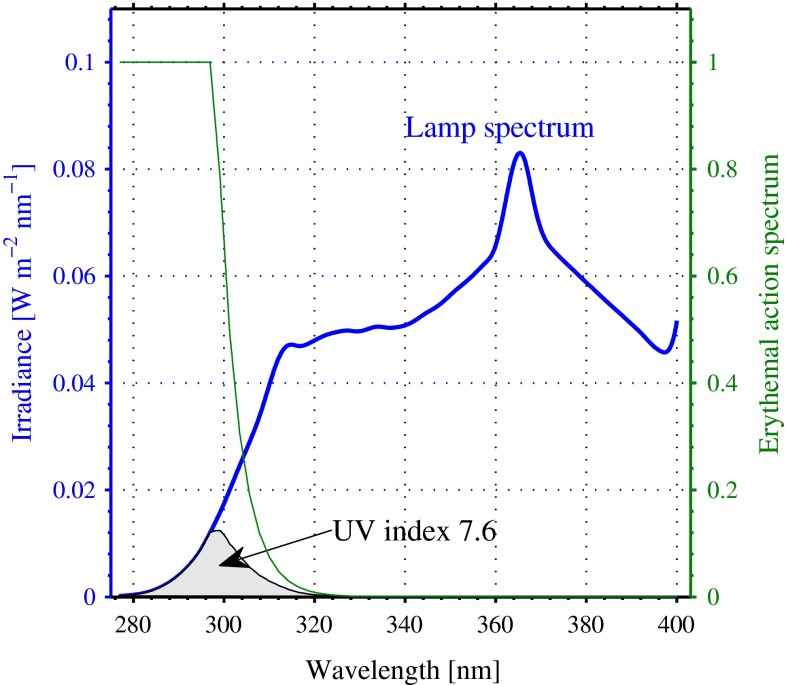
Spectral UV irradiance impinging on the algae inside the bottle (*blue curve*) together with the corresponding UV index computed as the integrated erythemal (*green curve*) weighted irradiance divided by 40. The in-bottle irradiance is inferred from a measured UV spectrum outside the bottle and the bottle transmittance

We define here four separate conditions on which fluorescence lifetime measurements were made:

1. Normal conditions: We used cultures of *Tetraselmis* sp. (Prasinophyceae), isolated from a fjord system outside the city of Bergen, Norway. The cells were batch-cultured (nonaxenic) at a temperature of $20\,^{\circ }\mathrm {C}$ in 500-ml glass bottles containing filtered 0.22 $\upmu \mathrm{m}$ pore size natural sea water supplemented with Conway nutrients (Walne 1966). The preconditioning light was provided by 40-W fluorescent light tubes, being regulated to give an incident irradiance of about 150 $\upmu \mathrm{mol}$ quanta $\mathrm{m^{-2}\,s^{-1}}$. The cultures were kept in darkness for at least 15 min before measurements were performed.
2. UV-stressed conditions: The algal cells were placed in a quartz glass bottle and kept for 2 h under the irradiation level given in Fig. 2 before they were moved to the microscope. The bottle was placed on a magnetic plate and a small magnet in the bottle ensured continuous stirring of the sample when irradiated. The light source was a series of lamp tubes providing UV-B, UV-A and PAR radiation, respectively. The effective UV dose rate is represented by a measured UV index of 7.6, which is comparable to the value found on a clear day, at the ocean surface, at noon, mid summer, at a latitude of about 50$^{\circ }$. Furthermore, we separated the UV-stressed conditions into two parts: (a) full dose, which is the dose represented in Fig. 2; (b) half dose, where the UV-B irradiance was halved, but otherwise the spectrum was as shown in Fig. 2.
3. Post UV-stressed conditions/re-dark adapted conditions: The quartz glass bottle was removed from the UV irradiation and kept in darkness for 24 h before the same sample was measured again.
4. DCMU (3-(3,4-dichlorophenyl)-1,1-dimethylurea) inhibited cells: A $10^{-5}\,\mathrm {M}$ concentration of DCMU was thoroughly mixed with a normal condition sample at a ratio of one part DCMU to nine parts cell culture. We allowed about 10 min for the DCMU to inhibit the cells before starting the measurements.

For all the samples, the procedure was to take a drop of the sample of the cells with a pipette and place it on a microscope slide, which was then covered by a 0.17-mm-thick glass before insertion onto the (inverted) microscope objective. All measurements were performed at room temperature (20 $^{\circ }\mathrm{C}$).

### Fluorometer measurements

An Aquapen-C AP-C 100 fluorometer (Photon Systems Instrument, Czech Republic) was used to measure the chlorophyll fluorescence. We extracted the minimum fluorescence intensity ${F}_\mathrm{o}$ and the maximum intensity ${F}_\mathrm{M}$ from the OJIP transient measurement (described in the introduction) and from these we calculated the variable fluorescence ${F}_\mathrm{V}={F}_\mathrm{M}-{F}_\mathrm{o}$, and the ratios ${F}_\mathrm{M}/{F}_\mathrm{o}$ and ${F}_\mathrm{V}/{F}_\mathrm{M}$. The Aquapen-C AP-C 100 induces fluorescence with blue + red light and detects fluorescence between 667 and 750 nm using bandpass filters.

### Lifetime measurements

To achieve a time resolution of 256 time channels, the scan resolution was set to 128 $\times$ 128 pixels. All measurements were performed with an excitation wavelength of 860 nm, which was determined the best for two-photon excitation of Chl *a*, which has an absorption maximum around 430 nm. To find the best focus, a 100-W ultra-high-pressure mercury-vapour (Hg) lamp integrated with the microscope were used to induce fluorescence in the algae cells. The detector interval was set to 650–720 nm to obtain a sufficiently strong fluorescence signal. All samples were scanned for 120 s to ensure that the photon count was high enough to obtain a reliable fluorescence lifetime decay curve, and at least five separate measurements from different parts of the sample were made so that the data were statistically significant.

### Data analysis

When lifetime data had been collected by the TCSPC module, the SPCImage software was used to calculate the decay matrix. The software automatically fits a decay curve to the collected photons to calculate a separate lifetime for each pixel in the resulting image. To indicate how well the decay curve fitted the lifetime function, a $\chi ^2$ value was calculated (1 indicates the best fit). After the decay matrix was calculated, both lifetime values, intensity and the relative amplitude of the lifetime components per pixel were exported. The exported data were treated in MatLab to calculate mean lifetimes and standard deviations.

## Results and discussion

All our measurements (normal, UV-stressed, post UV-stressed and DCMU-inhibited conditions) revealed two fluorescence lifetime components, and also all measurement conditions showed that the short component dominated with a relative amplitude between 80 and 90 %. As shown in Table 1, our measurements of Chl *a* under normal conditions resulted in two lifetime components, $\tau _1$ at 262 ps and $\tau _2$ at 728 ps. The relative amplitude of the short component was 87 %.

**Table 1:** The three first rows show chlorophyll *a* fluorescence lifetime data for normal conditions, UV-stressed conditions (2 h under full UV-B, UV-A and PAR) and post UV-stressed conditions

Condition	$\tau _1(\mathrm {ps})$	$\tau _2(\mathrm {ps})$	$a_1(\%)$
Normal	262 ± 31	728 ± 133	87 ± 5
UV stressed 2 h	389 ± 40	984 ± 145	85 ± 6
Post UV stressed 24 h	280 ± 42	886 ± 142	80 ± 5
UV stressed (reduced UV-B) 2 h	256 ± 23	750 ± 120	84 ± 5
UV stressed (reduced UV-B) 4 h	246 ± 25	823 ± 134	80 ± 7
DCMU inhibited	425 ± 46	1186 ± 321	82 ± 7

The fourth and fifth rows show chlorophyll *a* fluorescence lifetime data for half-dose UV-stressed conditions (2 and 4 h under 50 % UV-B in addition to full UV-A and PAR, respectively). The bottom row shows chlorophyll *a* fluorescence lifetime data for cells inhibited by DCMU. $\tau _1$ is the short lifetime component, $\tau _2$ is the long lifetime component, and $a_1$ is the relative amplitude of the short lifetime component. Uncertainties are calculated as one standard deviation

Due to the relatively long time scale (hours) in which the changes in fluorescence lifetime occur, it is most contiguous to attribute them to the type of NPQ called photoinhibitory quenching (qI), which is involved in long-term down-regulation of PSII. This classification is not well described in the literature; however it is believed that it is caused by a mix of photoprotection and photodamage (Müller et al. 2001). As photochemistry is down-regulated or shut down, the Chl fluorescence lifetime is known to increase. Chemically closing the reaction centres of the green microalgae *H. pluvialis* has been shown to result in an increase in lifetime from 250 ps under normal circumstances to 420 ps (Kristoffersen et al. 2012) for DCMU-inhibited cells, and our present measurements on DCMU-inhibited *Tetraselmis* cells showed a lifetime of 425 ps. Our results for UV-stressed conditions reveal a slightly shorter lifetime at 390 ps. This means that the removal of photochemistry as an energy sink likely explains most of the increase in lifetime.

Recovery from photoinhibition requires an increased synthesis of D1 protein (psbA gene), which has been shown to be accompanied by a decrease in the synthesis of the large subunit of rubisco. This could save resources such as the cellular nitrogen needed to activate the translation of chloroplast D1 mRNAs (Escoubas et al. 1995). It is also known that the NPQ involves de-epoxidation of xantophylls bound to LHCII trimers and minor antenna proteins, requiring specific polypeptides acting as efficient quenchers (Bassi and Caffarri 2000; Lavaud et al. 2003). We hypothesise that UV stress can cause a temporary disturbance in the link between proteins and PSII-antenna pigments. Interestingly, cells exposed to full-dose UV stress for both 1 h and 1 h 30 min did not show a significant change in fluorescence lifetime. This leads us to believe that rather than a gradual down-regulation of photochemistry when the cells are irradiated by UV, the photochemical apparatus functions at close to normal capacity until it suddenly (perhaps within minutes) breaks down. There is still a discrepancy between the fluorescence lifetime of the DCMU-inhibited cells and the UV-stressed cells, so probably the electron transport between PSII and PSI is not completely shut down in the UV-stressed situation (which is the case for DCMU-inhibited cells).

We also measured the lifetimes of cells having been UV stressed with only 50 % (of full dose) UV-B irradiation in addition to full UV-A and PAR light. After both 2 and 4 h of irradiation, the lifetimes did not change significantly, as shown in Table 1. From this we concluded that at least on this timescale (a few hours) and UV-A radiation levels, UV-B is the only type of radiation (within our irradiance spectrum) that is significantly damaging to the algal cells and the cause of the increase in the Chl *a* fluorescence lifetime. This can at least partially be explained by the formation of damaging oxygen radicals and DNA degradation. The controlling role of DNA for many cellular processes makes it difficult to foresee the total consequence of DNA damage within the cell, but it is probably responsible for a number of inhibitory effects (Mitchell and Karentz 1993). Among these is protein synthesis, which is a prerequisite for D1 repair. Such a response has been demonstrated for *Tetraselmis* sp., where the repair of damaged D1 protein slowed down during UV-B exposure (Ma et al. 2012). Although the fluorescence lifetime did not change when we used the 50 % UV-B dose, it is likely that the LHCII detached from the PSII reaction centre and moved to the PSI reaction centre. This leaves PSII as the main site of the observed UV-B effect. Most likely the D1 protein and/or other proteins in the PSII core antenna are the sensitive targets for full UV-B radiation. In accordance with this, it was previously found that UV-B exposure led to breakage of polypeptide bindings in the PSII core of the green alga *Chlorella salina* and the prymnesiophyte *Dicrateria inornata* (Vimalabai and Kulandaivelu 2002). Recovery from the damaging light stress was obtained by transferring the culture to darkness for 24 h. This resulted in the Chl *a* fluorescence lifetimes returning to normal condition values and shows that more or less complete repairs can occur on a time scale of hours.

**Fig. 3:**
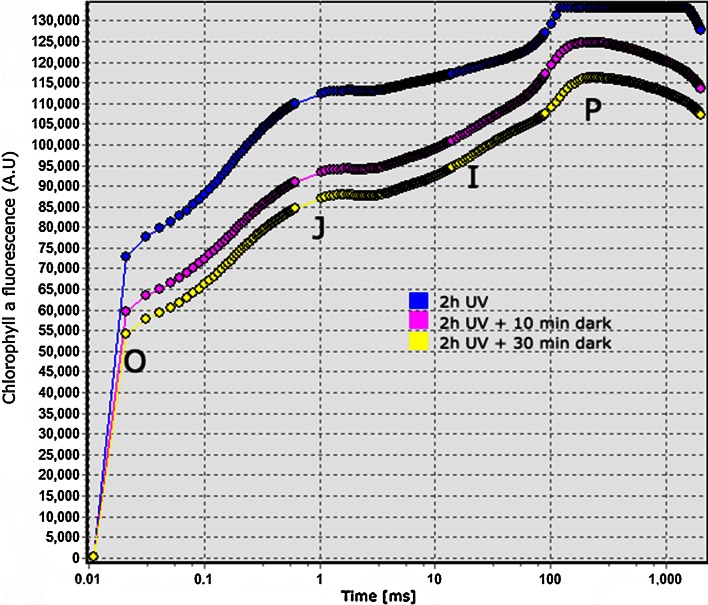
Chlorophyll *a* fluorescence transient OJIP for *Tetraselmis* cells after 2 h of the full UV dose (*blue*) and the same sample after 10 (*pink*) and 30 (*yellow*) min in darkness, respectively

In addition to fluorescence lifetime measurements, we performed a measurement of the chlorophyll *a* fluorescence transient OJIP for both normal and UV-stressed cells. Figure 3 shows the OJIP transient for cells directly after 2 h of UV irradiation and the same sample after 10 and 30 min of dark adaptation, respectively. The ratios are shown in Table 2 for normal dark-adapted cells, cells under 2 h of UV irradiation and the UV-stressed cells after 10 and 30 min of dark adaptation, respectively, as well as after 24 h of recovery in darkness.

**Table 2:** The ratios ${F}_\mathrm{M}/{F}_\mathrm{o}$ and ${F}_\mathrm{V}/{F}_\mathrm{M}$ calculated from OJIP fluorescence transient measurements of chlorophyll *a* for normal cells, cells exposed to 2 h of full-dose UV radiation, dark-adapted cells for 10 and 30 min after 2 h of full-dose UV, respectively, as well as cells that recovered in darkness for 24 h

Cell condition	${F}_\mathrm{M}/{F}_\mathrm{o}$	${F}_\mathrm{V}/{F}_\mathrm{M}$
Normal dark-adapted cells	3.86	0.74
2 h full-dose UV irradiation	1.67	0.4
2 h UV + 10-min dark adaptation	1.92	0.48
2 h UV + 30-min dark adaptation	1.96	0.49
24 h recovery in darkness	3.66	0.69

The ratio of maximum fluorescence over minimum fluorescence ${F}_\mathrm{M}/{F}_\mathrm{o}$ shows that the value for *Tetraselmis* (3–4) is significantly lower compared to normal values for plant leaves (5–6). After full-dose UV the ratio is very low, at 1.67, which means that after UV stress, a large portion of the reaction centres is already closed, damaged or destroyed. However, after only 10 min of further dark adaptation, the ratio increases to a value of 1.92, and after 24 h the ratio returned to 3.66, which is very close to the value for normal conditions. The ratio of variable fluorescence over maximum fluorescence ${F}_\mathrm{V}/{F}_\mathrm{M}$ is often used as a stress indicator, with values for normal cells usually in the range 0.6–0.7 and decreasing typically with 50 % after severe stress (both nutrient and UV stress show similar behaviour) (Parkhill et al. 2001; Guihéneuf et al. 2010; White et al. 2010). Our results show a normal cell value of 0.74, as shown in Table 2, and after 2 h of full-dose UV irradiance the ratio decreased to 0.4. After the cells were dark adapted the ratio started to increase again to 0.48 after 10 min, and 0.49 after 30 min, indicating that D1 protein synthesis begins almost immediately after the cells are dark adapting; however it is a slow process since there was almost no difference in the ratio ${F}_\mathrm{V}/{F}_\mathrm{M}$ from 10 to 30 min. After 24 h of recovery in darkness, the ratio was 0.69, almost at the normal value. These fluorescence transient results support the fluorescence lifetime findings, showing that after 24 h of recovery in darkness, the cells return to almost the same values measured for normal dark-adapted cells, meaning that the damage caused by the UV radiation is probably almost repaired, but not completely.

**Fig. 4:**
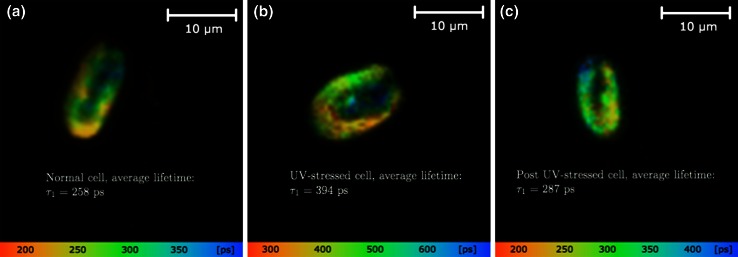
Chlorophyll fluorescence lifetime images of three separate *Tetraselmis* cells for three different conditions: **a** normal conditions; **b** after 2 h of UV stress; **c** kept in darkness overnight

In Fig. 4, the fluorescence lifetime is imaged to show how the lifetime varies in different parts of the cell. It is clear that the lifetime is non-uniform throughout the cell; however it remains similar within subregions of the cell. Also, the outer subregions have a shorter lifetime compared to the inner regions. For the particular cell that was used for the image of a normal cell, the average lifetime was $\tau =258\,\mathrm {ps}$, while an average of all the normal cells was calculated to be $\tau =262\,\mathrm {ps}$. For the UV-stressed cell, the lifetime was about twice as long, although it seems that there were to a much larger degree both short and long lifetimes present in the same subregions—or that the subregions in a way dissolved into a mix of short and long lifetimes. Interestingly, for the re-dark adapted cell, the same seems to be the case. This shows that some pigments are not affected by the UV stress.

Our results show that for the cell as a whole, the longest fluorescence lifetimes are located at the anterior part of the *Tetraselmis* cell, irrespective of light treatment. There are four chloroplast lobes pointing towards the anterior end of the cell, and this is also where the flagellar root is positioned (Fig. 4, see also Fig. 1 for comparison). Common for *Tetraselmis* species is that the nucleus is positioned in the centre of the cell (uncoloured in Fig. 4), while the pyrenoid is found in the posterior part surrounded by thylakoids (Chengwu and Hongjun 2002). Another characteristic feature of the *Tetraselmis* anatomy is the location of the mitochondria, which are found close to the flagellar root at the anterior end of the cell (Van Den Hoek 1995). Such organisation is favourable with respect to the energy demand of the flagella. The calcium-sensitive and contractile phosphoprotein centrin constitutes the main part of the flagellar root in *Tetraselmis striata* and its phosphorylation is coupled to flagellar root contraction (Martindale and Salisbury 1990). Root contraction is supposed to be related to the inflow of Ca$^{2+}$ ions (Salisbury 1982). A key factor in this context is ATP (Mitchell et al. 2005). It has been discussed whether the ATP can diffuse fast enough from the mitochondria in the cytoplasm and into the root to satisfy the P-demand here. The situation will be still worse for diffusion of ATP from the chloroplast stroma situated farther away. It has been pointed out that unless the ATP diffusion rates exceed the hydrolysis rates within the root, there will be a steep gradient in ATP, and the distal parts of the flagella will be starved for ATP, with the conclusion that there is a need for additional ATP synthesis in the flagellar root system to support motility in *Chlamydomonas reinhardtii* (Mitchell et al. 2005). Due to the fact that *Tetraselmis* has four flagella, two more than *Chlamydomonas*, there is reason to believe that there is a higher demand for orthophosphate-P within the flagellar root of *Tetraselmis*. We therefore hypothesise that competition for orthophosphate-P in the anterior end of the cell among photophosphorylation in the chloroplast lobe, oxidative phosphorylation in the mitochondria and phosphorylation within the flagellar root results in photosynthetic P-limitation. By slowing down photophosphorylation, accumulation of H$^{+}$ in the thylakoid lumen will appear. The consequence will be reduced electron transport from PSII to PSI and thereby lowered photochemical quenching, resulting in a longer Chl *a* fluorescence lifetime.

## Conclusion

In vivo measurements of the green microalga *Tetraselmis* showed that the main chlorophyll *a* fluorescence lifetime $\tau _1$ (contributing 80–90 % of the relative intensity) increases significantly from 262 ps under normal conditions to 389 ps after being exposed to full-dose UV raditation for 2 h. Cells infiltrated by DCMU, halting photosynthesis completely, had a fluorescence lifetime $\tau _1= 425$ ps, indicating that the UV stress has a serious negative effect on the photochemistry of the cells. UV stress where UV-B was reduced to half irradiance did not significantly affect the fluorescence lifetime, indicating that UV-B is the only radiation able to damage the cells on a timescale of up to 4 h. After dark adapting the cells exposed to a full 2-h dose of UV for 24 h, the fluorescence lifetime was measured to be 280 ps, almost at normal levels, suggesting that the cells were able to repair the damage to a high degree on a relatively short timescale. OJIP fluorescence transient measurements showed that the stress-indicator ratio ${F}_\mathrm{V}/{F}_\mathrm{M}$ decreased from 0.74 for normal conditions to 0.4 for 2-h full UV and returned to 0.69 after recovering in darkness for 24 h, thereby supporting the results from the lifetime measurements. Imaging of the cells for the fluorescence lifetime showed a more heterogeneous distribution of lifetimes after UV stress compared to normal conditions, and the cells after 24 h of darkness showed a similar distribution to that of the normal cells.

## References

[CR1] Ahn TK, Avenson TJ, Ballottari M, Cheng Y-C, Niyogi KK, Bassi R, Fleming GR (2008). Architecture of a charge-transfer state regulating light harvesting in a plant antenna protein. Science.

[CR2] Amarnath K, Zaks J, Park SD, Niyogi KK, Fleming GR (2012). Fluorescence lifetime snapshots reveal two rapidly reversible mechanisms of photo‘protection in live cells of Chlamydomonas reinhardtii. Proc Natl Acad Sci.

[CR3] Bassi R, Caffarri S (2000). Lhc proteins and the regulation of photosynthetic light harvesting function by xanthophylls. Photosynth Res.

[CR4] Björkman O, Demmig B (1987). Photon yield of O$$_{2}$$ evolution and chlorophyll fluorescence characteristics at 77 k among vascular plants of diverse origins. Planta.

[CR5] Broess K, Borst J, van Amerongen H (2009). Applying two-photon excitation fluorescence lifetime imaging microscopy to study photosynthesis in plant leaves. Photosynth Res.

[CR6] Chengwu Z, Hongjun H (2002). Taxonomy and ultrastructure of five species of *Tetraselmis* (Prasinophyceae) isolated from China seas. Acta Oceanol Sin.

[CR7] Demmig-Adams B, Adams WW (1996). The role of xanthophyll cycle carotenoids in the protection of photosynthesis. Trends Plant Sci.

[CR8] Dring M, Makarov V, Schoschina E, Lorenz M, Lüning K (1996). Influence of ultraviolet-radiation on chlorophyll fluorescence and growth in different life-history stages of three species of *Laminaria* (phaeophyta). Mar Biol.

[CR9] Dring MJ, Wagner A, Boeskov J, Lüning K (1996). Sensitivity of intertidal and subtidal red algae to UVA and UVB radiation, as monitored by chlorophyll fluorescence measurements: Influence of collection depth and season, and length of irradiation. Eur J Phycol.

[CR10] Escoubas JM, Lomas M, LaRoche J, Falkowski PG (1995). Light intensity regulation of cab gene transcription is signaled by the redox state of the plastoquinone pool. Proc Natl Acad Sci.

[CR11] Falkowski PG, Kolber Z (1995). Variations in chlorophyll fluorescence yields in phytoplankton in the world oceans. Aust J Plant Physiol.

[CR12] Gilmore AM, Hazlett TL, Govindjee (1995) Xanthophyll cycle-dependent quenching of photosystem II chlorophyll a fluorescence: formation of a quenching complex with a short fluorescence lifetime. Proc Natl Acad Sci 92(6):2273–2277 10.1073/pnas.92.6.2273 PMC42466 11607518

[CR13] Gómez I, Figueroa F (1998). Effects of solar UV stress on chlorophyll fluorescence kinetics of intertidal macroalgae from southern spain: a case study in *Gelidium* species. J Appl Phycol.

[CR14] Guihéneuf F, Fouqueray M, Mimouni V, Ulmann L, Jacquette B, Tremblin G (2010). Effect of UV stress on the fatty acid and lipid class composition in two marine microalgae *Pavlova lutheri* (Pavlovophyceae) and *Odontella aurita* (Bacillariophyceae). J Appl Phycol.

[CR15] Heber U (2002). Irrungen, wirrungen? the mehler reaction in relation to cyclic electron transport in C3 plants. Photosynth Res.

[CR16] Hessen D, De Lange H, Van Donk E (1997). UV-induced changes in phytoplankton cells and its effects on grazers. Freshw Biol.

[CR17] Holzinger A, Karsten U, Lütz C, Wiencke C (2006). Ultrastructure and photosynthesis in the supralittoral green macroalga *Prasiola crispa* from Spitsbergen (Norway) under UV exposure. Phycoligia.

[CR18] Holzinger A, Lütz C (2006). Algae and UV irradiation: Effects on ultrastructure and related metabolic functions. Micron.

[CR19] Holzwarth AR, Miloslavina Y, Nilkens M, Jahns P (2009). Identification of two quenching sites active in the regulation of photosynthetic light-harvesting studied by time-resolved fluorescence. Chem Phys Lett.

[CR20] Iwai M, Yokono M, Inada N, Minagawa J (2010). Live-cell imaging of photosystem II antenna dissociation during state transitions. Proc Natl Acad Sci.

[CR21] Jahns P, Holzwarth AR (2012). The role of the xanthophyll cycle and of lutein in photoprotection of photosystem II. Biochim Biophys Acta (BBA) Bioenerg.

[CR22] Johnson MP, Ruban AV (2009). Photoprotective energy dissipation in higher plants involves alteration of the excited state energy of the emitting chlorophyll(s) in the light harvesting antenna II (LHCII). J Biol Chem.

[CR23] Kautsky H, Hirsch A (1931). Neue versuche zur kohlensäureassimilation. Naturwissenschaften.

[CR24] Kolber Z, Falkowski PG (1993). Use of active fluorescence to estimate phytoplankton photosynthesis in situ. Limnol Oceanogr.

[CR25] Krieger-Liszkay A (2005). Singlet oxygen production in photosynthesis. J Exp Bot.

[CR26] Kristoffersen AS, Svensen Ø, Ssebiyonga N, Erga SR, Stamnes JJ, Frette Ø (2012). Chlorophyll a and NADPH fluorescence lifetimes in the microalgae *Haematococcus pluvialis* (chlorophyceae) under normal and astaxanthin-accumulating conditions. Appl Spectrosc.

[CR27] Lakowicz JR (1999). Principles of Fluorescence Spectroscopy.

[CR28] Lavaud J, Rousseau B, Etienne A-L (2003). Enrichment of the light-harvesting complex in diadinoxanthin and implications for the nonphotochemical fluorescence quenching in diatoms. Biochemistry.

[CR29] Li Z, Wakao S, Fischer BB, Niyogi KK (2009). Sensing and responding to excess light. Ann Rev Plant Biol.

[CR30] Ma Z, Helbling E, Li W, Villafañe V, Gao K (2012). Motility and photosynthetic responses of the green microalga *Tetraselmis subcordiformis* to visible and UV light levels. J Appl Phycol.

[CR31] Manton I, Parke M (1965). Observations on the fine structure of two species of Platymonas with special reference to flagellar scales and the mode of origin of the theca. J Mar Biol Assoc UK.

[CR32] Martindale V, Salisbury J (1990). Phosphorylation of algal centrin is rapidly responsive to changes in the external milieu. J Cell Sci.

[CR33] Meindl U, Lütz C (1996). Effects of UV irradiation on cell development and ultrastructure of the green alga *Micrasterias*. J Photochem Photobiol B: Biol.

[CR34] Miller R, Wu G, Deshpande RR, Vieler A, Gärtner K, Li X, Moellering ER, Zäuner S, Cornish AJ, Liu B, Bullard B, Sears BB, Kuo M-H, Hegg EL, Shachar-Hill Y, Shiu S-H, Benning C (2010). Changes in transcript abundance in *Chlamydomonas reinhardtii* following nitrogen deprivation predict diversion of metabolism. Plant Physiol.

[CR35] Miloslavina Y, Grouneva I, Lambrev PH, Lepetit B, Goss R, Wilhelm C, Holzwarth AR (2009). Ultrafast fluorescence study on the location and mechanism of non-photochemical quenching in diatoms. Biochim Biophys Acta (BBA) Bioenerg.

[CR36] Mitchell BF, Pedersen LB, Feely M, Rosenbaum JL, Mitchell DR (2005). ATP production in *Chlamydomonas reinhardtii* flagella by glycolytic enzymes. Mol Biol Cell.

[CR37] Mitchell D, Karentz D, Young A, Moan J, Björn L, Nultsch W (1993). The induction and repair of DNA photodamage in the environment. Environmental UV Photobiology.

[CR38] Müller MG, Lambrev P, Reus M, Wientjes E, Croce R, Holzwarth AR (2010). Singlet energy dissipation in the photosystem II light-harvesting complex does not involve energy transfer to carotenoids. Chem Phys Chem.

[CR39] Müller P, Li X-P, Niyogi KK (2001). Non-photochemical quenching. A response to excess light energy. Plant Physiol.

[CR40] Nilkens M, Kress E, Lambrev P, Miloslavina Y, Müller M, Holzwarth AR, Jahns P (2010). Identification of a slowly inducible zeaxanthin-dependent component of non-photochemical quenching of chlorophyll fluorescence generated under steady-state conditions in *Arabidopsis*. Biochim Biophys Acta (BBA) Bioenerg.

[CR41] Niyogi KK (1999). Photoprotection revisited: Genetic and molecular approaches. Ann Rev Plant Physiol Plant Mol Biol.

[CR42] Papageorgiou G, Govindjee (2004). Chlorophyll a fluorescence: a signature of photosynthesis.

[CR43] Parkhill J, Maillet G, Cullen J (2001). Fluorescence-based maximal quantum yield for PSII as a diagnostic of nutrient stress. J Phycol.

[CR44] Roach T, Krieger-Liszkay A (2012). The role of the PsbS protein in the protection of photosystems I and II against high light in *Arabidopsis thaliana*. Biochim Biophys Acta (BBA) Bioenerg.

[CR45] Rozema J, Björn L, Bornman J, GaberŠČik A, Häder D-P, Trošt T, Germ M, Klisch M, Gröniger A, Sinha R, Lebert M, He Y-Y, Buffoni-Hall R, de Bakker N, van de Staaij J, Meijkamp B (2002). The role of UV-B radiation in aquatic and terrestrial ecosystemsG an experimental and functional analysis of the evolution of UV-absorbing compounds. J Photochem Photobiol B: Biol.

[CR46] Rozema J, van de Staaij J, Björn LO, Caldwell M (1997). UV-B as an environmental factor in plant life: stress and regulation. Trends Ecol Evol.

[CR47] Ruban AV, Berera R, Ilioaia C, Van Stokkum IH, Kennis JT, Pascal AA, Van Amerongen H, Robert B, Horton P, Van Grondelle R (2007). Identification of a mechanism of photoprotective energy dissipation in higher plants. Nature.

[CR48] Ruban AV, Johnson MP, Duffy CD (2012). The photoprotective molecular switch in the photosystem II antenna. Biochim Biophys Acta (BBA) Bioenerge.

[CR49] Salisbury J (1982). Calcium-sequestering vesicles and contractile flagellar roots. J Cell Sci.

[CR50] Schreiber U, Bilger W, Neubauer C, Schulze E-D, Caldwell MM (1995). Chlorophyll fluorescence as a nonintrusive indicator for rapid assessment of in vivo photosynthesis. Ecophysiology of Photosynthesis.

[CR51] Shapira M, Lers A, Heifetz P, Irihimovitz V, Barry Osmond C, Gillham N, Boynton JE (1997). Differential regulation of chloroplast gene expression in *Chlamydomonas reinhardtii* during photoacclimation: light stress transiently suppresses synthesis of the rubisco LSU protein while enhancing synthesis of the PS II D1 protein. Plant Mol Biol.

[CR52] Smith GJ, Alberts RS (1991). Characterization of photosystem I-associated polypeptides from the chlorophyll b-rich alga *Tetraselmis spp.* (pleurastrophyceae) and other chlorophyte algae. J Phycol.

[CR53] Song X-Z, Gibbs S (1995). Photosystem I is not segregated from photosystem II in the green alga *Tetraselmis subcordiformis*. Protoplasma.

[CR54] Stirbet A, Govindjee (2011). On the relation between the Kautsky effect (chlorophyll a fluorescence induction) and photosystem II: Basics and applications of the OJIP fluorescence transient. J Photochem Photobiol B: Biol 104(1 2):236 – 257.** (Special Issue on Recent Progress in the Studies of Structure and Function of Photosystem II)** 10.1016/j.jphotobiol.2010.12.010 21295993

[CR55] Taiz L, Zeiger E (2006). Plant Physiology.

[CR56] Van Den Hoek, C. (1995). *Algae*. University Press

[CR57] Vimalabai C, Kulandaivelu G (2002). Effects of prolonged UV-B enhanced fluorescent radiation on some marine microalgae. Biol Plant.

[CR58] Walne PR (1966) Experiments in the large-scale culture of the larvae of *Ostrea edulis* L

[CR59] White S, Anandraj A, Bux F (2010). PAM fluorometry as a tool to assess microalgal nutrient stress and monitor cellular neutral lipids. Bioresour Technol.

[CR60] Yamamoto H (2006). Functional roles of the major chloroplast lipids in the violaxanthin cycle. Planta.

